# Feature fusion network based on few-shot fine-grained classification

**DOI:** 10.3389/fnbot.2023.1301192

**Published:** 2023-11-09

**Authors:** Yajie Yang, Yuxuan Feng, Li Zhu, Haitao Fu, Xin Pan, Chenlei Jin

**Affiliations:** College of Information Technology, Jilin Agriculture University, Changchun, China

**Keywords:** few-shot classification, fine-grained classification, similarity measurement, inter-class distinctiveness, intra-class compactness

## Abstract

The objective of few-shot fine-grained learning is to identify subclasses within a primary class using a limited number of labeled samples. However, many current methodologies rely on the metric of singular feature, which is either global or local. In fine-grained image classification tasks, where the inter-class distance is small and the intra-class distance is big, relying on a singular similarity measurement can lead to the omission of either inter-class or intra-class information. We delve into inter-class information through global measures and tap into intra-class information via local measures. In this study, we introduce the Feature Fusion Similarity Network (FFSNet). This model employs global measures to accentuate the differences between classes, while utilizing local measures to consolidate intra-class data. Such an approach enables the model to learn features characterized by enlarge inter-class distances and reduce intra-class distances, even with a limited dataset of fine-grained images. Consequently, this greatly enhances the model's generalization capabilities. Our experimental results demonstrated that the proposed paradigm stands its ground against state-of-the-art models across multiple established fine-grained image benchmark datasets.

## 1. Introduction

Deep learning models have achieved remarkable results in the realm of visual recognition, spanning tasks like image and text classification (LeCun et al., 2015; Dvornik et al., 2019; Lin et al., 2020). However, in real-world settings, the efficacy of these models often hinges on the presence of vast amounts of training data (Simonyan and Zisserman, 2015; Gu et al., 2017; Lin et al., 2017b). For certain categories, only a handful of labeled instances might be available. Yet, humans can rapidly acquire knowledge with limited data (Li et al., 2020). Drawing inspiration from this human-centric learning approach, the concept of few-shot learning (Jankowski et al., 2011) has been introduced to align machine learning more closely with human cognition.

In recent years, a plethora of methods have emerged in the field of few-shot learning. Broadly, these can be categorized into meta-learning methods (Lake et al., 2017; Ravi and Larochelle, 2017; Nichol et al., 2018; Rusu et al., 2019) and metric learning methods (Vinyals et al., 2016; Li et al., 2017; Snell et al., 2017). Meta-learning-based approaches concentrate on sampling learners from the distribution for each task or episode. They execute the optimizer or conduct several unfolded weight updates in parallel to adapt the model specifically for the task at hand. On the other hand, metric learning methods prioritize embedding both support and query samples into a common feature space to gauge feature similarity (Vinyals et al., 2016). Among these, metric learning stands out for its simplicity, ease in introducing new categories, and capability for incremental learning. It has shown exemplary performance on fine-grained images (Lin et al., 2017a; Li et al., 2019a).

Fine-grained few-shot learning (Chen et al., 2020; Shermin et al., 2021) frequently utilizes datasets such as Stanford-Cars (Wang et al., 2021), Stanford-Dogs (Krause et al., 2013), FGVC-Aircraft (Khosla et al., 2013), and CUB-200-2011 (Maji et al., 2013). Each of these datasets consists of numerous subcategories, with only a handful of images per subclass. Given the marked similarities between each class within fine-grained datasets, the crux of fine-grained image classification revolves around pinpointing local areas exhibiting nuanced differences (Wah et al., 2011). Consequently, efficiently detecting foreground objects and identifying critical local area information has emerged as a pivotal challenge in the realm of fine-grained image classification algorithms (Zhang et al., 2017; Li et al., 2019b; Ma et al., 2019).

**Figure 1:**
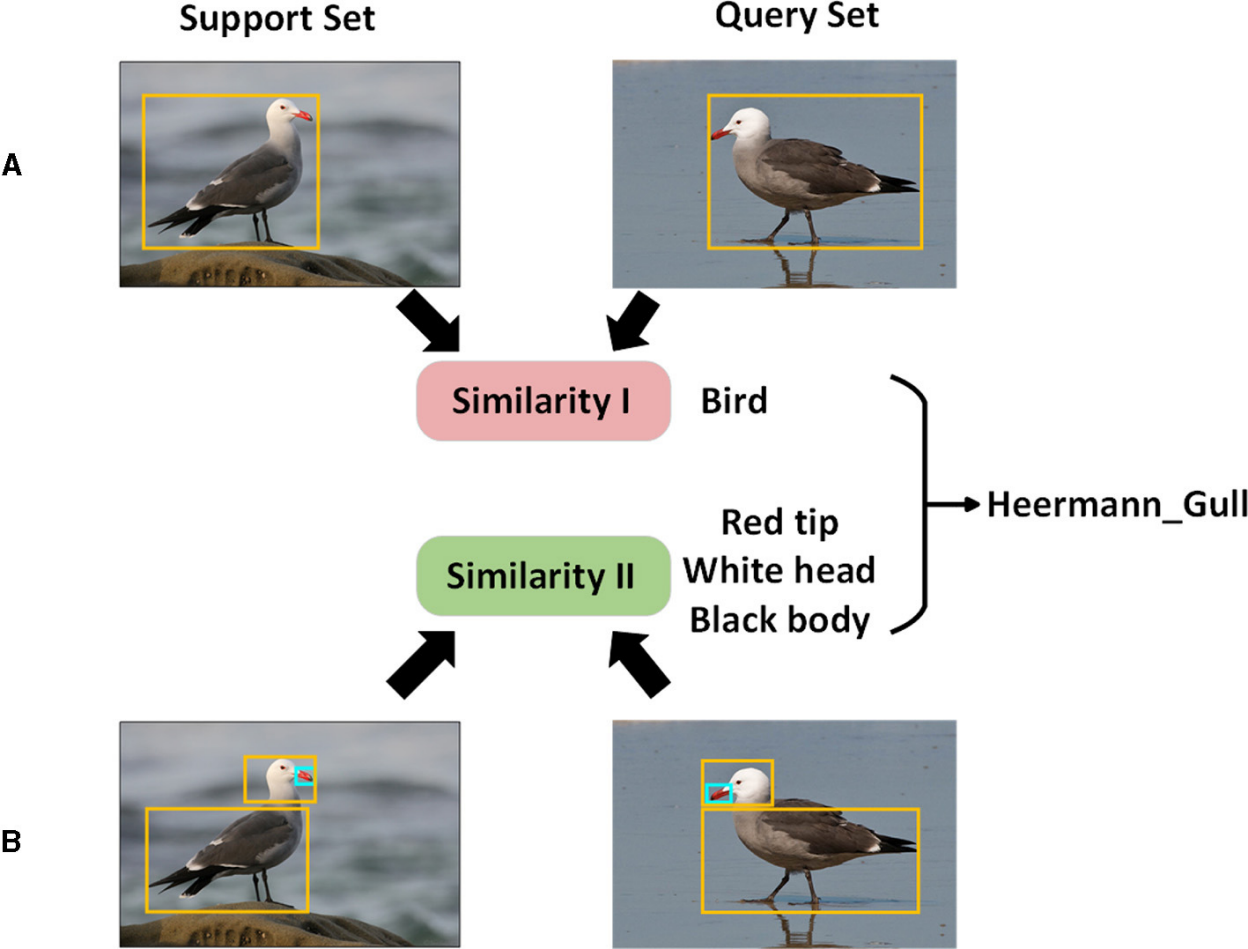
Motivation diagram. **(A)** In the diagram, the exploration of global information aims to maximize the separation distance between different classes; **(B)** in the diagram, the exploration of local information directs the network to concentrate on crucial local regions and assigns varying weights to make intra-class information more compact; ultimately, global and local information are combined for class determination.

Few-shot metric learning methods that are easy to operate typically employ a single similarity measure. Traditional metric learning techniques, like matching networks and relation networks, often depend on entire image features for recognition. This approach, however, is not particularly effective for fine-grained image classification tasks where the inter-class distances are narrow and the intra-class distances are broad. DN4 and TDSNet (Chang et al., 2020) have introduced deep local descriptors, shifting the focus toward local feature information. Nevertheless, relying on a single similarity measure can introduce particular similarity biases, especially when dealing with limited training data. This can undermine the model's generalization capability. As illustrated in Figure 1, merging global and local features can amplify the inter-class differences and condense the intra-class variances, enhancing accuracy in fine-grained image classification.

**Figure 2:**
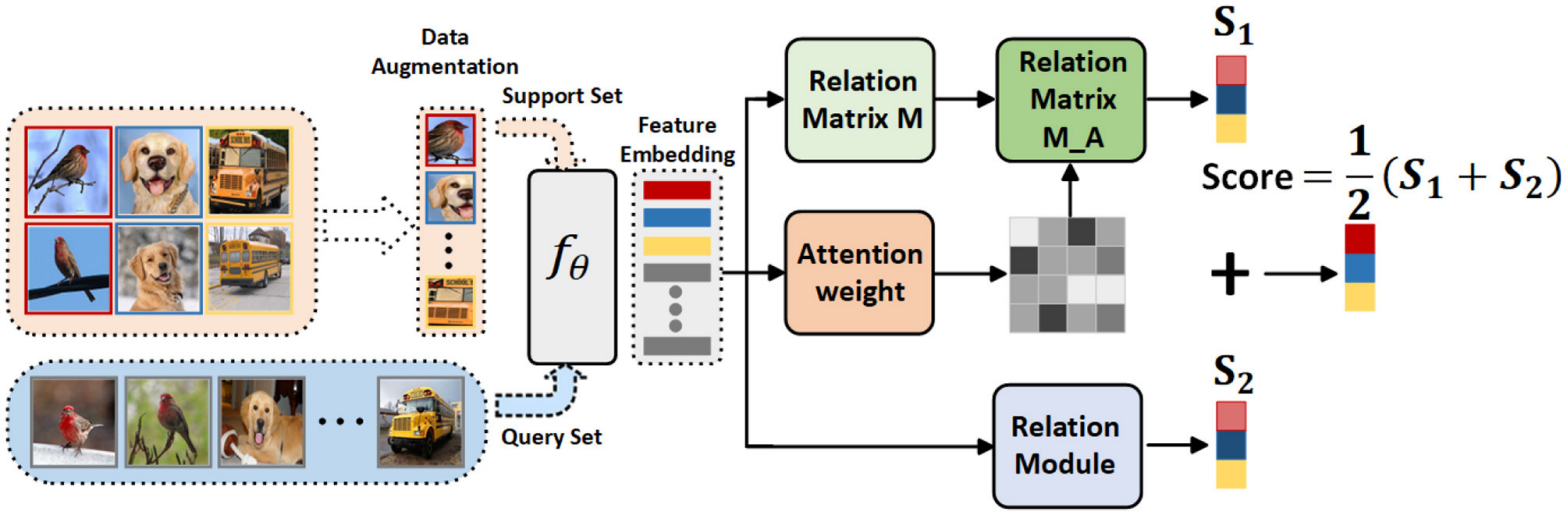
Schematic of the FFSNet framework. The support set initially passes through a data augmentation module and is then combined with the query set in the embedding module. Following the fusion of global information extraction and local important area feature weighting, the resulting features are utilized for classification purposes.

In this paper, we introduce a Feature Fusion Similarity Network designed to assess global and local similarities within each task generated by the meta-training dataset. It fully leverages the global invariant features and local detailed features present in the images. The Feature Fusion Similarity Network comprises three modules, as illustrated in Figure 2. The initial module is a convolution-based embedding module responsible for generating feature information for both query and support images and subsequently assessing similarity through global and local modules. During the meta-training phase, the total loss is computed as the sum of the global and local losses. Extensive experiments have been conducted to showcase the performance of the proposed Feature Fusion Similarity Network. Our contributions can be summarized as follows: (1) We introduce a novel few-shot fine-grained framework that incorporates global and local features, enabling the extraction of fine-grained image feature information during the meta-learning training process. (2) The fusion of global and local features not only explicitly constructs crucial connections between different parts of fine-grained objects but also implicitly captures fine-grained details with discriminative characteristics. (3) We conducted comprehensive experiments on four prominent fine-grained datasets to demonstrate the effectiveness of our proposed approach. The results of these experiments affirm the high competitiveness of our proposed model.

## 2. Related work

Fine-grained image recognition has consistently ranked among the most active research domains in Fine-Grained Visual Recognition (FGVR), presenting a set of significant challenges. The strong performance of deep learning techniques on large datasets with labeled information has been noteworthy. For instance, Qi et al. (2022) employed a bilinear network to capture local distinctions among different subordinate classes, enhancing discriminative capabilities. Fu et al. (2017) demonstrated the feasibility of employing an optimization scheme to train attention proposal networks and region-based classifiers when dealing with related tasks. Lin et al. (2015) introduced the Diversified Visual Attention Network (DVAN), with a particular emphasis on diversifying attention mechanisms to better capture crucial discriminative information.

Building upon the foundation of fine-grained image recognition, few-shot fine-grained image recognition has garnered significant attention (Zhao et al., 2017). This approach enables models to recognize new fine-grained categories with minimal labeled information. Few-shot fine-grained image recognition can be categorized into methods based on meta-learning and methods based on metric learning.

Meta-learning-based methods: In Rusu et al. (2019), a model adapts to new episodic tasks by generating parameter updates through a recursive meta-learner. MAML (Finn et al., 2017) and its variants (Lake et al., 2017; Ravi and Larochelle, 2017) have demonstrated that optimizing the parameters of the learner model enables it to quickly adapt to specific tasks. However, Lake et al. (2017) pointed out that while these methods iteratively handle samples from all classes in task updates, they often struggle to learn effective embedding representations. To address this issue, one approach involves updating the weights only for the top layer and applying them to the “inner loop.” Specifically, the top-layer weights can be initialized by sampling from a generative distribution conditioned on task samples and then pretraining on visual features during the initial supervised phase. In contrast, metric-learning-based methods have achieved considerable success in learning high-quality features.

Metric-learning-based methods: Metric learning methods primarily focus on learning a rich similarity metric. In Snell et al. (2017), the concept of episode training was introduced in few-shot learning, while prototype networks (Li et al., 2017) determine the category of a query image by comparing its distance to class prototypes in the support set, inspired by Wei et al. (2019). Relation networks (Sung et al., 2018) utilize neural networks with cascaded feature embeddings to assess the relationship between each query-support set pair. DN4 (Li et al., 2019a) employs K-nearest neighbors to create an image-to-class search space using local representations. Lifchitz et al. (2019) directly predicts the classification of each local representation and computes the loss. Our method combines global and local metrics. Unlike relation networks, we integrate local information while considering global relationships. Through local measurements, we effectively compare two objects rather than two images, which enhances the ease and effectiveness of the process. By merging global and local features, our algorithm has demonstrated satisfactory results.

## 3. Approach

### 3.1. Problem definition

Few-shot classification is often defined as an *N* − *way* *K* − *shot* classification problem. An *N* − *way* *K* − *shot* classification problem refers to a scenario where a small dataset *S*, also known as a support set, contains limited labeled information. The support set comprises *N* categories of images, with each category containing *K* labeled sample images, where the value of *K* can vary from 1 to 10 samples. In a given query set *Q*, each sample is an unlabeled instance awaiting classification. The objective of few-shot classification is to perform classification on the unlabeled samples in the query set *Q*, utilizing the limited information available in the support set *S*.

To address this issue, Vinyals et al. (2016) introduced an episode training approach. Given a task *T* = {*D*_*s*_, *D*_*q*_}, it comprises a support set $D_{s}={\left\{x_{i^{\prime }}^{s}y_{i}^{s}\right\}}_{i=1}^{N_{s}}$ (where *x*_*i*_ represents an image in the support set, and *y*_*i*_ represents the label of the image) and a query set $D_{q}={\left\{x_{i}^{q}\right\}}_{i=1}^{N_{q}}$. The support set consists of a total of *N*_*S*_ = *N* × *K* labeled sample images, while the query set *D*_*q*_ contains *N*_*q*_ unlabeled sample images. In each episode, training is carried out by iteratively constructing the support set $D_{s}={\left\{x_{i^{\prime }}^{s}y_{i}^{s}\right\}}_{i=1}^{N_{s}}$ and the query set $D_{q}={\left\{x_{i}^{q}\right\}}_{i=1}^{N_{q}}$ through random sampling. This same sampling pattern must be adhered to during the testing process. The trained model can then recognize each sample in the query set *Q* by utilizing the constructed support set *S*, where *C*_*train*_ ∩ *C*_*test*_ = ∅.

### 3.2. Feature fusion similarity network

The overall model framework is depicted in Figure 2. Firstly, data augmentation is applied to the support set, which is then fed along with the query set into the embedding module *f*_θ_. This embedding module can either be a simple convolutional layer or a ResNet (He et al., 2016), used for extracting feature information to obtain image feature vectors. These image feature vectors serve as inputs for the Relation Matrix *M* Module, Attention Weight Module, Relation Matrix *M*_*A* Module, and Relation Module. The Relation Matrix *M* module constructs a local description space and calculates the similarity between the query image and the corresponding local region in the support set. The Attention Weight Module reweights the locally significant areas and, utilizing a *K*-Nearest-Neighbors classifier (*K* − *NN* for short), calculates the similarity of the query image to each category in the support set, effectively reducing noise. The Relation Matrix *M*_*A* Module serves as a refinement of the Attention Weight Module. Its purpose is to assess the correctness of the locally important areas and to fuse and calculate similarities with the feature map obtained from the Relation Matrix *M* Module. The Relation Module concatenates the feature vector corresponding to each query set image with the feature vector of each image in the support set to calculate their similarity. These two similarities are then combined to obtain class information.

#### 3.2.1. Data augmentation module

In the data augmentation module, we discovered that applying extensive transformations to the original image led to a decrease in classification accuracy during training and resulted in substantial information loss in smaller data domains. As a result, we opted for horizontal flipping enhancements and adjustments to image contrast processing, which perturb the data without sacrificing information, thereby ensuring consistency between training and testing.

#### 3.2.2. Feature embedding module

**Figure 3:**
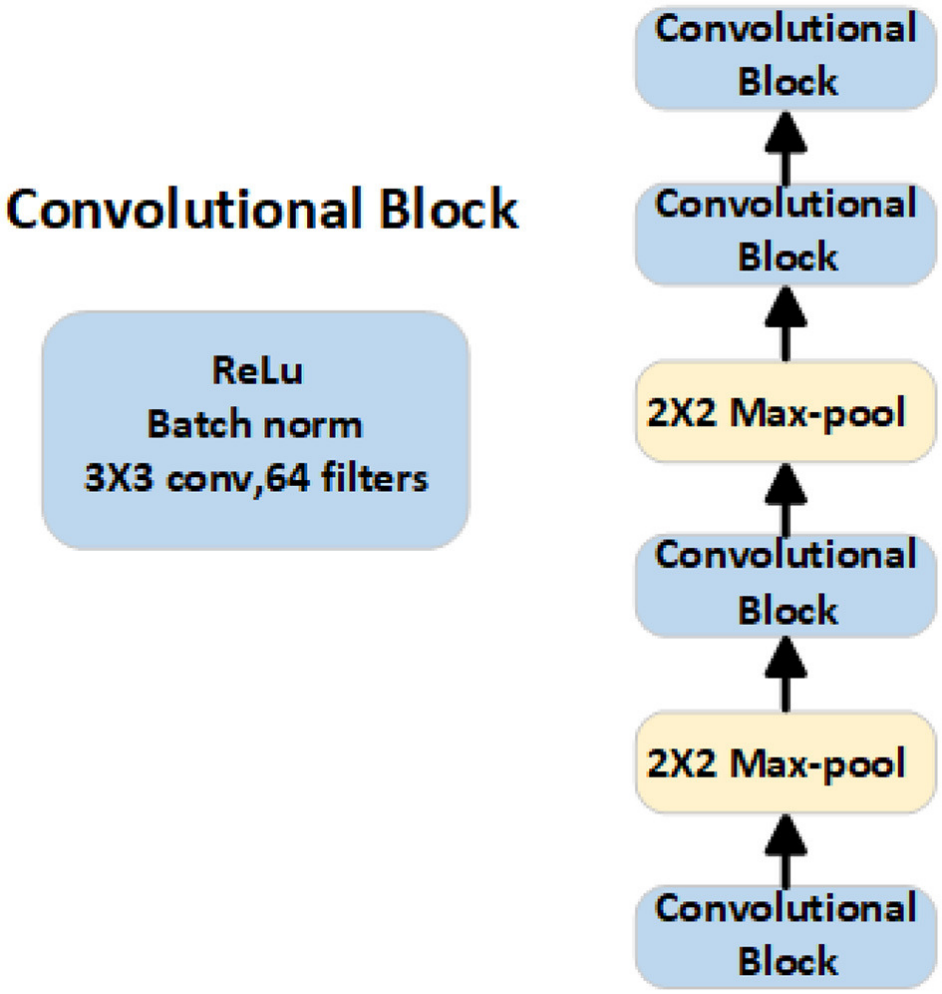
Structural framework of the embedding module, which is responsible for extracting feature information from both the support set and the query set.

We chose four convolutional blocks as the embedding module of the network to extract feature information. Each convolutional block consists of a 3 × 3 convolution with 64 filters, followed by batch normalization and ReLU activation function, as shown in Figure 3.

The extracted *f_θ_*(*S*) and *f_θ_*(*Q*) from the embedding module are represented as follows:


(1) $$\begin{array}{l} \text{Input}\left(S\right):\left(N,K,C,H,W\right)\to f_{\theta }\left(S\right):\left(N\cdot K,C_{1},H_{1},W_{1}\right) \end{array}$$

(2) $$\begin{array}{l} \text{Input}\left(Q\right):\left(N,K,C,H,W\right)\to f_{\theta }\left(Q\right):\left(K,C_{1},H_{1},W_{1}\right) \end{array}$$

where *N*·*K* represents the total number of samples in the support set, with *N* classes and *K* samples per class. *N* represents the number of classes, *K* represents the number of samples in the support set and query set, and *C*, *H*, *W* respectively represent the number of input channels, width, and height.

#### 3.2.3. Relation matrix *M* module

Recent research on DN4 and TDSNet has shown that features based on local descriptors are more distinctive than global features. Specifically, local descriptors have the ability to capture subtle details in the local areas of an image. After being extracted by the Feature Embedding module into $f_{\theta }\left(\cdot \right)\epsilon R^{H\times W\times C}$, it can be represented as *m* = (*H* × *W*) individual d-dimensional local descriptors. The support set can be represented as $f_{\theta }\left(S\right)\epsilon R^{N\times m\times C}$, and similarly, the query set is $f_{\theta }\left(Q\right)\epsilon R^{m\times C}$. Next, we construct the relationship matrix *M* for local similarity calculation.


(3) $$\begin{array}{l} M=COS\left(f_{\theta }\left(Q\right),f_{\theta }\left(S\right)\right) \end{array}$$

where *COS*(·) represents the cosine similarity, and each row of the relationship matrix *M* represents the similarity between a local area of the query image and each local area of the support set.

#### 3.2.4. Attention weight module

The *M*^*A*^ module takes the query set features and support set features as input, and directly generates spatial attention to locate the support class objects in the query image, thereby creating an attention map on the query image. In this module, we pass the *f*_θ_(·) extracted by the Feature Embedding module through a 1 × 1 convolution block. The purpose of this step is to reweight the local areas. When calculating local similarity, if there are multiple duplicate areas in the query image that overlap with the support set, it can lead to misjudgment during discrimination. Therefore, this module disperses attention and focuses on the relatively smaller parts in the local descriptor, effectively reweighting them. By employing the Relation Matrix *M* Module method, an Attention Matrix *M*^*A*^ is constructed. Subsequently, the minimum value of the Attention Matrix is obtained through the *TOP* − *K* of the *K* − *NN* (*K*-Nearest Neighbors) algorithm to suppress noise.


(4) $$\begin{array}{l} M^{A}=I\left(M\right) \end{array}$$

(5) $$I(x)=\{\begin{array}{ll} x, & \text{if }x>\beta \\ 0, & {\text{otherwise}}_{,} \end{array}$$

where *β* is selected by the *K* − *NN* (*K*-Nearest Neighbors) algorithm based on the first three minimum values of the relation matrix *M*.

#### 3.2.5. Relation matrix *M*_−_*A* module

We obtain the element-wise multiplication between the feature matrix and the relationship matrix *M*, which is crucial for classification, using the weight matrix *M*^*A*^. Finally, the local similarity score between the query image *X*_*q*_ and the support class *X*_*s*_ can be computed by applying the attention map to the similarity matrix *M*, as shown below:


(6) $$\begin{array}{l} S_{1}=M_{-}A=\frac{1}{HW}\overset{HW}{\underset{i=1}{\sum }}\overset{HW}{\underset{j=1}{\sum }}{\left(M^{A}\cdot M\right)}_{i,j,} \end{array}$$

where *H* and *W* represent the row and column indices of the matrix.

#### 3.2.6. Relation module

In the relationship network, the relationship module is used to concatenate the feature vector *f*_θ_(*x*_*q*_) corresponding to the query set image with the feature information *f*_θ_(*x*_*n*_) corresponding to each image in the support set.


(7) $$\begin{array}{l} S_{2}=g_{\varphi }\left(\left[f_{\theta }\left(x_{n}\right)||f_{\theta }\left(x_{q}\right)\right]\right),\;n=1,\cdots \left(D_{n}\right) \end{array}$$

where || represents the concatenation operator. The similarity module *g*_φ_ consists of two 3 × 3 convolution blocks, each followed by a 2 × 2 max-pooling layer and a fully connected layer.

The final formula for the overall prediction score is:


(8) $$\begin{array}{l} S_{total}=\frac{1}{2}\left(S_{1}+S_{2}\right) \end{array}$$

where *S*_1_ represents the score of the Relation Matrix *M*_−_*A* Module, and *S*_2_ represents the score of the Relation Module.

#### 3.2.7. Loss function

In the Feature Fusion Similarity Network, we obtain two prediction results, *y*^1^ and *y*^2^, through the local module and global module, respectively. Then, we calculate the losses *l*_1_ and *l*_2_ between the two prediction results and the true values.


(9) $$\begin{array}{l} L_{1}=\overset{N}{\underset{j}{\sum }}{\left(y_{q,j}^{1}-y_{q,j}\right)}^{2},q=1,\cdots \left(D_{q}\right) \end{array}$$

(10) $$\begin{array}{l} L_{2}=\overset{N}{\underset{j}{\sum }}{\left(y_{q,j}^{2}-y_{q,j}\right)}^{2},q=1,\cdots \left(D_{q}\right) \end{array}$$

where *y*^1^ represents the predicted label and *y* represents the true label.

Because the network architecture we have designed combines local and global information, after obtaining the loss *L*_1_ of the local module and the loss *L*_2_ of the global module separately, we need to add them together to calculate the overall loss of the model:


(11) $$\begin{array}{l} L_{total}=L_{1}+L_{2} \end{array}$$

Where *L*_1_ represents the loss function of the Relation Matrix *M*_−_*A* Module, and *L*_2_ represents the loss function of the Relation Module.

## 4. Experiments

### 4.1. Experiments settings

The experimental datasets used in this paper consist of four common datasets for few-shot classification. Among them, three datasets are frequently employed in fine-grained image classification in the context of few-shot learning: Stanford-Dogs, Stanford-Cars, and CUB-200-2011. To assess the generalizability of our model, we also conducted experiments on the widely used few-shot classification dataset Mini-ImageNet.

The Stanford-Dogs dataset comprises 120 categories of dogs, totaling 20,580 images. This dataset divides the 120 categories into training, validation, and test sets, consisting of 60, 30, and 30 classes, respectively. The Stanford-Cars dataset consists of 196 car classes, with a total of 16,185 images. It divides these 196 classes into training, validation, and test sets, consisting of 98, 49, and 49 classes, respectively. CUB-200-2011 includes 200 bird species and has a total of 11,788 images. This dataset divides its 200 classes into training, validation, and test sets, comprising 100, 50, and 50 classes, respectively. Mini-ImageNet is a subset of ImageNet (Deng et al., 2009), containing 100 classes, with 600 images per class, totaling 60,000 images. Mini-ImageNet's 100 classes are divided into training, validation, and test sets, consisting of 64, 16, and 20 classes, respectively, following the method described in Ravi and Larochelle (2017). The total number of samples in the training, validation, and test sets of the above datasets can be found in Table 1.

**Table 1:** Data set partition table.

**Datasets**	**Divide**	**Number of classes**	**Sample number**
	Train	60	10,337
Stanford-Dogs	Val	30	5,128
	Test	30	5115
	Train	98	8,203
Stanford-Cars	Val	49	4,004
	Test	49	3,978
	Train	100	4,719
CUB-200-2011	Val	50	4,715
	Test	50	2,953
	Train	64	38,400
Mini-ImageNet	Val	16	9,600
	Test	20	12,000

#### 4.1.1. Evaluation metrics

We reported the average accuracy (%) for 600 randomly generated episodes, along with the 95% confidence interval on the test set, following the approach commonly used in most methods (Jankowski et al., 2011; Nichol et al., 2018; Xu et al., 2022). Our model was trained end-to-end, without any pre-training process.

#### 4.1.2. Implementation details

We conducted experiments on four datasets: Stanford-Dogs, Stanford-Cars, CUB-200-2011, and Mini-ImageNet, using two settings, namely 5-Way 1-shot and 5-Way 5-shot. There were 15 query samples per class. The input image size was resized to 84 × 84. We randomly sampled 60,000 and 40,000 tasks under the 1-shot and 5-shot experimental settings, respectively, to iteratively train the model. During the training process, we utilized the Adam optimizer (Kingma and Ba, 2017) to optimize the model parameters with mean squared error loss, setting an initial learning rate of 0.001 and a weight decay rate of 0.

### 4.2. Performance comprison

#### 4.2.1. Few-shot fine-grained image classification

We conducted classification experiments using the proposed method on three fine-grained image classification datasets: Stanford-Dogs, Stanford-Cars, and CUB-200-2011, with 5-Way 1-shot and 5-Way 5-shot experiments. We compared the experimental results with the current mainstream methods, and the results are presented in Table 2.

**Table 2:** Comparison with typical FSL methods on three fine-grained.

**Model**	**5-Way accuracy (** ***%*** **)**					
	**Stanford-Dogs**		**Stanford-Cars**		**CUB-200-2011**	
	**1-shot**	**5-shot**	**1-shot**	**5-shot**	**1-shot**	**5-shot**
Matching Net (Vinyals et al., 2016)	45.05 ± 0.66	59.60 ± 0.73	45.29 ± 0.82	64.00 ± 0.74	55.65 ± 0.38	72.60 ± 0.45
Prototype Net (Snell et al., 2017)	39.05 ± 0.66	60.23 ± 0.22	36.38 ± 0.52	63.84 ± 0.85	51.52 ± 0.95	70.21 ± 0.38
Relation Net (Sung et al., 2018)	47.11 ± 0.90	64.56 ± 0.74	45.83 ± 0.86	68.01 ± 0.78	62.67 ± 0.98	76.94 ± 0.66
MAML (Finn et al., 2017)	46.67 ± 0.87	62.56 ± 0.80	48.37 ± 0.81	65.41 ± 0.77	55.92 ± 0.94	72.09 ± 0.76
PCM (Wei et al., 2019)	28.78 ± 0.95	46.92 ± 0.85	29.63 ± 0.65	52.28 ± 0.78	42.10 ± 0.35	62.48 ± 0.35
CovaMNet (Li et al., 2019b)	49.11 ± 0.56	63.04 ± 0.76	56.65 ± 0.86	69.56 ± 0.78	52.42 ± 0.76	63.76 ± 0.64
DN4 (Li et al., 2019a)	45.41 ± 0.76	63.51 ± 0.62	57.84 ± 0.80	**87.47** **±** **0.47**	46.84 ± 0.81	74.92 ± 0.64
BSNet (Li et al., 2021)	50.68 ± 0.56	67.93 ± 0.75	54.39 ± 0.92	72.37 ± 0.77	65.89 ± 0.46	78.48 ± 0.65
TDSNet (Qi et al., 2022)	52.48 ± 0.87	66.45 ± 0.49	57.35 ± 0.91	73.64 ± 0.72	67.34 ± 0.85	79.38 ± 0.59
Our	**53.98** **±** **0.96**	**70.85** **±** **0.75**	**58.32** **±** **0.62**	75.68 ± 0.76	**68.30** **±** **0.90**	**80.64** **±** **0.64**

Bold values represents the maximum value in each column.

From Table 2, it can be observed that our proposed method achieves the highest classification accuracy in the 1-shot experiments on the datasets Stanford-Dogs, Stanford-Cars, and CUB-200-2011. This is attributed to our method assigning higher weights to significant local information while simultaneously integrating global information to enhance accuracy. In the 5-shot experiment on Stanford-Cars, our classification results are slightly lower than those of DN4. However, when compared to some classic few-shot models such as Matching Net, Prototype Net, Relation Net, and MAML, there is a noticeable improvement. In the 1-shot experiments, we see improvements of 8.93%, 14.93%, 6.87%, and 7.31% on Stanford-Dogs, and 13.03%, 21.94%, 12.49%, and 9.95% on Stanford-Cars. Similarly, in the 1-shot experiment on CUB-200-2011, we observe improvements of 12.65%, 16.78%, 5.63%, and 12.38%. In the 5-shot experiments, our method also demonstrates improvements of 11.25%, 10.62%, 6.29%, and 8.29% on Stanford-Dogs, and 11.68%, 11.84%, 7.67%, and 10.27% on Stanford-Cars. Additionally, in the 5-shot experiment on CUB-200-2011, there are improvements of 8.04%, 10.43%, 3.7%, and 8.55%.

#### 4.2.2. Few-shot image classification

To further assess the generalization performance of the method proposed in this paper, we conducted 5-Way 1-shot and 5-Way 5-shot classification experiments on the Mini-ImageNet dataset. We compared the experimental results with mainstream few-shot image classification methods to validate the generalization performance of our proposed method. The results are presented in Table 3.

**Table 3:** The accuracy of few-shot image classification on the Mini-ImageNet dataset.

**Model**	**Type**	**5-Way accuracy (** ***%*** **)**	
		**1-shot**	**5-shot**
Meta-Learner LSTM (Ravi and Larochelle, 2017)	Meta-learning	43.44 ± 0.77	60.60 ± 0.71
MAML (Finn et al., 2017)	Meta-learning	46.47 ± 0.82	62.71 ± 0.71
Matching net (Vinyals et al., 2016)	Metric learning	48.14 ± 0.23	63.48 ± 0.66
Prototype net (Snell et al., 2017)	Metric learning	44.42 ± 0.84	64.24 ± 0.72
Relation net (Sung et al., 2018)	Metric learning	49.33 ± 0.85	65.44 ± 0.69
Matching nets FCE (Vinyals et al., 2016)	Metric learning	43.56 ± 0.96	55.31 ± 0.73
GNN (Garcia and Bruna, 2018)	Metric learning	50.33 ± 0.36	65.23 ± 0.86
Our	Metric learning	**52.37** **±** **0.78**	**68.19** **±** **0.95**

Bold values represents the maximum value in each column.

In Table 3, meta-learning disregards the training complexity of the model and the challenges associated with model convergence, leading to lower overall classification accuracy. In comparison to MAML, our method exhibits improvements of 5.9 and 5.48% in the 1-shot and 5-shot experiments, respectively. Furthermore, when compared to Meta-Learner LSTM, it demonstrates improvements of 8.93 and 7.59% in the 1-shot and 5-shot experiments, respectively. Moreover, in comparison to the metric-based approaches such as Matching Net, Prototype Net, Relation Net, GNN, and Matching Nets FCE as shown in Table 3, our method outperforms them in terms of classification performance in both the 1-shot and 5-shot experiments on Mini-ImageNet. This underscores that our proposed method exhibits superior classification performance on the Mini-ImageNet dataset.

## 5. Analysis

### 5.1. Ablation experiment

To comprehensively validate the effectiveness of the proposed Feature Fusion Similarity Network, we conducted an ablation experiment on the CUB-200-2011 dataset using the Baseline, which consists of the feature embedding module and relationship module presented in this paper. As shown in Table 4, several key modules were added incrementally to the baseline model. Firstly, the addition of the aug (data augmentation module) led to an increase in the accuracy of our model by 3.35 and 2.93% for 1-shot and 5-shot experiments, respectively. This improvement is mainly attributed to the data augmentation module enhancing the model's robustness, reducing its sensitivity to images, increasing the training data, improving the model's generalization ability, and mitigating sample imbalance. Subsequently, by incorporating the LS (local similarity module), we observed a notable increase in accuracy of 5.23 and 5.64% for 1-shot and 5-shot experiments, respectively. This demonstrates that relying solely on the global module makes it challenging to detect subtle local information. The inclusion of the local module makes the system more sensitive to certain fine-grained local details. Finally, we verified the effectiveness of the att (attention weight module), which improved the accuracy in 1-shot and 5-shot experiments by 8.98 and 7.42%, respectively. The att module reweights important local areas, suppresses noise, and enhances the model's performance.

**Table 4:** Results of ablative experiments on CUB-200-2011 datasets.

**Model**	**5-Way accuracy (** ***%*** **)**	
	**1-shot**	**5-shot**
Baseline	59.32 ± 0.71	73.22 ± 0.14
Baseline + aug	62.67 ± 0.98	76.15 ± 0.66
Baseline + aug + LS	64.55 ± 0.89	78.86 ± 0.37
Baseline + aug + LS + att	**68.30** **±** **0.90**	**80.64** **±** **0.64**

aug, data augmentation module; LS, local similarity module; att, attention weight moudle. Bold values represents the maximum value in each column.

### 5.2. Case study

**Figure 4:**
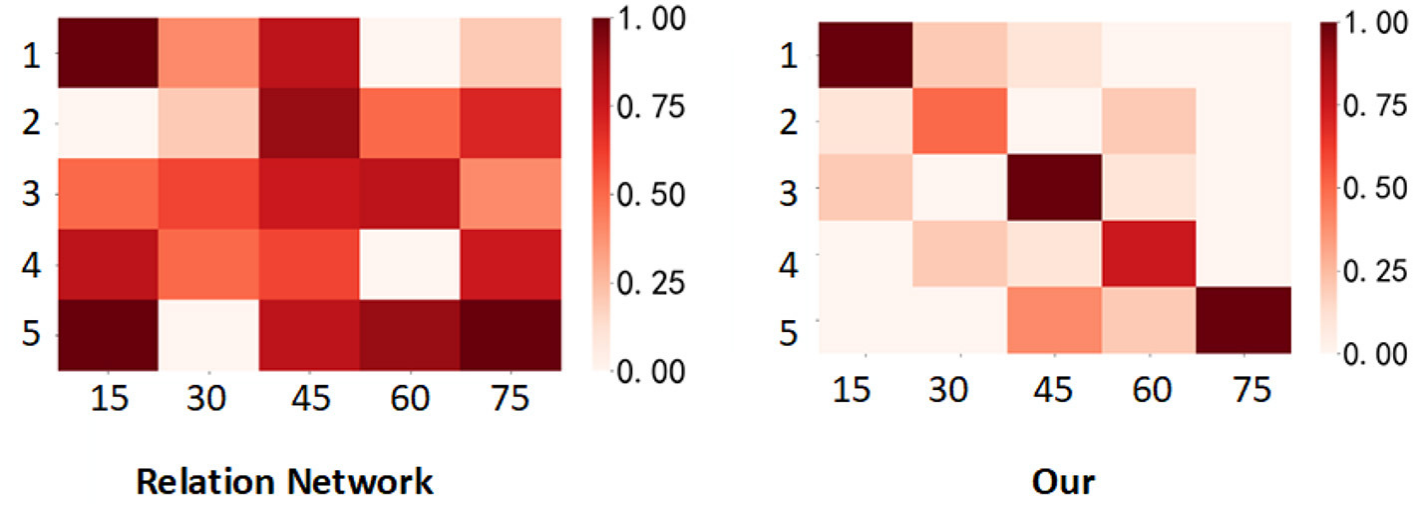
Visualization of the similarity scores predicted by the Relation Network and the network proposed in this paper on the CUB-200-2011 dataset is presented. In each matrix, the horizontal axis represents 15 query samples from each of the five classes, totaling 75 samples. The vertical axis represents the five classes in the task. The deeper the color, the higher the similarity.

From Figure 4, it is evident that the color change trend on the CUB-200-2011 dataset indicates that our proposed network model exhibits better classification accuracy in the experiment compared to the original relation network. The relation network's inter-class discriminability and intra-class compactness on fine-grained datasets need improvement, which results in numerous misjudged areas in the measurement results and subsequently leads to poor outcomes. In contrast, our network demonstrates a high adaptability to fine-grained datasets and excels in fine-grained classification. This improvement enhances both inter-class discriminability and intra-class compactness, contributing to its superior performance.

**Figure 5:**
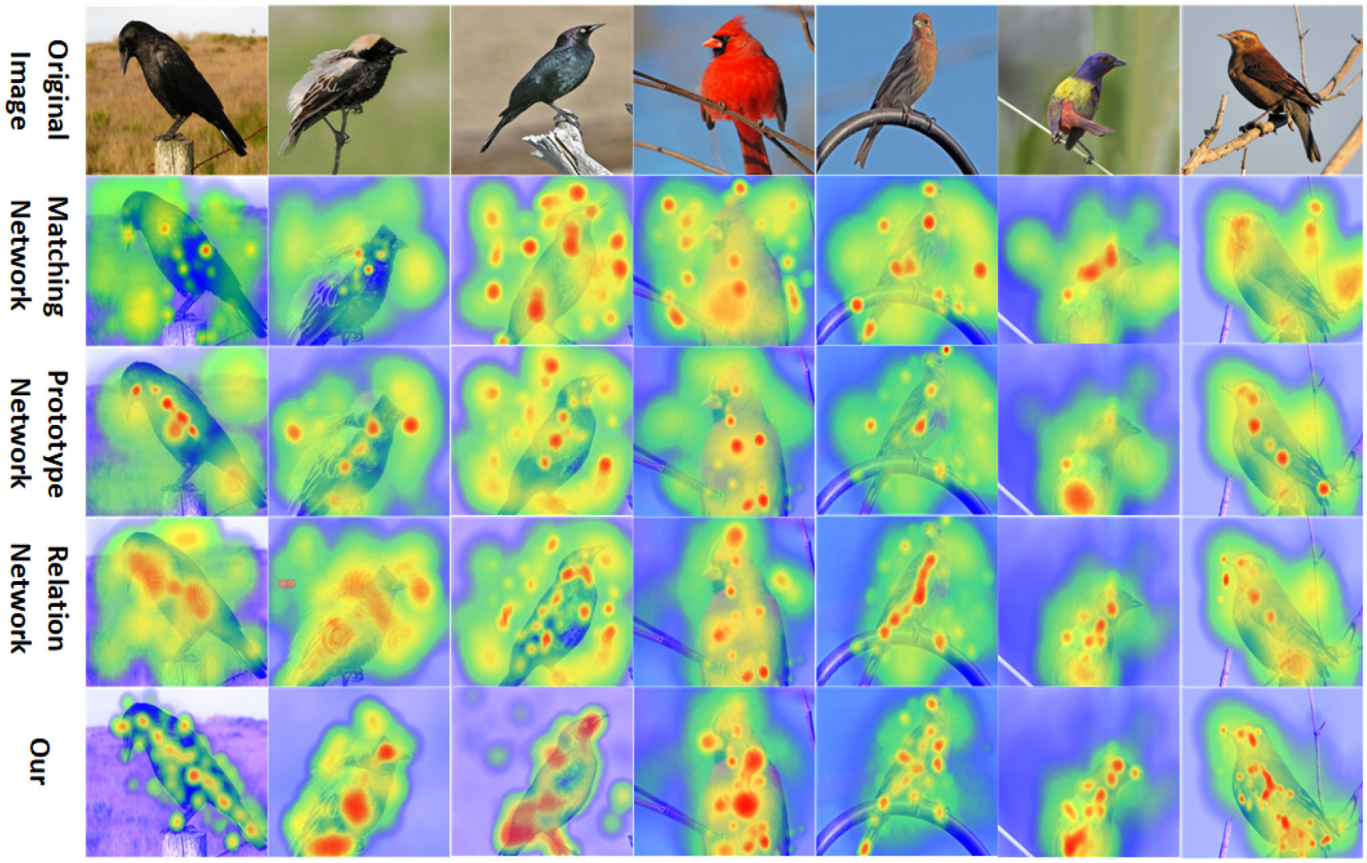
Feature visualization of the Matching Network, Prototype Network, Relation Network, and our Network on the CUB-200-2011 dataset (the redder the area, the more class-discriminative it is).

We visualized important areas in the original images using the gradient-based Grad-CAM (Selvaraju et al., 2017) technique on the CUB-200-2011 dataset. Nine original images were randomly selected from the CUB-200-2011 test dataset, and these selected images were resized to match the size of the output features from the embedding module. Subsequently, Grad-CAM images were generated using the Matching Network, Prototype Network, Relation Network, and our Network. As shown in Figure 5, it is evident from the images that the class-discriminative areas in our Network are primarily concentrated on the target object, whereas other methods also exhibit more class-discriminative areas distributed in the background. Therefore, our model is more efficient in focusing on the target object and reducing background interference.

## 6. Conclusion

The article introduces a network that integrates both global and local information, termed the Feature Fusion Similarity Network. This network comprises image data augmentation, feature embedding modules, and both global and local metric modules. By amalgamating global and local insights, it becomes possible to discern regions from various angles, which in turn amplifies classification accuracy. Thorough experimental evidence reveals that our approach showcases competitive performance on fine-grained image datasets when juxtaposed with other mainstream methodologies. Moving forward, our objective is to further refine the object features within images to optimize the model's classification capabilities.

## Data availability statement

The raw data supporting the conclusions of this article will be made available by the authors, without undue reservation.

## Author contributions

YY: Data curation, Methodology, Software, Writing—original draft, Writing—review & editing. YF: Software, Writing—original draft. LZ: Writing—original draft, Writing—review & editing. HF: Writing—original draft. XP: Writing—review & editing. CJ: Writing—original draft.
